# Lipid ciliology: specialized ciliary membrane lipids in physiology and disease

**DOI:** 10.3389/fcell.2026.1893158

**Published:** 2026-07-21

**Authors:** Alamgir Hasan, Tomoka Morita, Moe Hirosawa, Nao Kitamura, Ryoya Murakami, Masamichi Mikuni, Daigo Kobayashi, Takeshi Itabashi, Tatsuo Miyamoto

**Affiliations:** 1 Department of Cellular and Molecular Physiology, Graduate School of Medicine, Yamaguchi University, Ube, Yamaguchi, Japan; 2 Division of Advanced Genome Editing Therapy Research, Research Institute for Cell Design Medical Sciences, Yamaguchi University, Ube, Yamaguchi, Japan

**Keywords:** cholesterol, ciliopathies, lipid ciliology, phosphoinositides, primary cilia

## Abstract

Primary cilia are the microtubule-based sensory organelles. The unique lipid makeup of the ciliary membrane strictly controls their signaling ability, to orchestrate tissue formation and homeostasis. Emerging evidence has demonstrated that lipids play important roles in cilia formation and cilia-related signaling, solidifying the previously proposed concept of “lipid ciliology.” The ciliary membrane exhibits a highly specialized lipid composition, including enrichment in cholesterol, sphingolipids, and specific phosphoinositides, compared with the surrounding plasma membrane. Recent studies have revealed that cholesterol and phosphoinositides function together to regulate ciliary homeostasis, protein trafficking, and signal transduction. A growing spectrum of ciliopathies, including polycystic kidney disease, retinal degeneration, cerebellar hypoplasia, and metabolic disorders, can be caused by dysregulation of lipid metabolism and lipid-modifying enzymes through impaired cilia-related signaling. Moreover, defects in cholesterol biosynthesis or intracellular lipid transport contribute to various ciliopathies, such as Smith–Lemli–Opitz syndrome and Zellweger spectrum disorders. In this review, we summarize recent advances in lipid ciliology, focusing on the underlying molecular mechanisms of ciliary cholesterol-dependent signaling in ciliopathies for emerging therapeutic strategies.

## 1 Introduction

Cilia are microtubule-based protrusions that develop on the surface of vertebrate cells during the G0 phase (Amack, 2022; Li and Ran, 2024). There are two main types of cilia found in the body: motile cilia and primary cilia. Motile cilia are most frequently assembled as multiple autonomously but synchronously moving organelles originating from a single cell and performing physiological functions by generating local fluid flow. For example, the fluid flow generated by motile cilia in the airway epithelium is essential for inhaled particles. In contrast, primary cilia are generally non-motile sensory organelles, formed as a single structure per cell. Primary cilia harbor various receptors and channels and function as cellular sensors that detect mechanical and fluidic information from the extracellular environment for embryonic development and tissue homeostasis (Nigg and Raff, 2009; Anvarian et al., 2019; Mill et al., 2023). In this review, we will primarily focus on primary cilia and discuss the roles of lipids in their physiological functions, a research paradigm collectively referred to as “lipid ciliology” and defined here as the study of ciliary membrane lipids, their spatiotemporal dynamics, and their disruption in ciliopathies.

Morphologically, primary cilia possess four core structures: A basal body i.e, a transformed mother centriole, the transition zone (TZ), the axoneme composed of a microtubule array, and the ciliary membrane (Anvarian et al., 2019; Sun et al., 2019; Moran et al., 2024). Germline mutations and somatic mutations of genes involved in the function and structure of primary cilia cause ciliopathies, a group of rare genetic disorders characterized by clinical symptoms depending on the mutated gene, including polycystic kidney disease, retinopathy, polydactyly, cerebellar hypoplasia, and visceral heterotaxy (Hildebrandt et al., 2011; Reiter and Leroux, 2017). Over the past two decades, typical ciliopathies such as Joubert syndrome (JS), Meckel–Gruber syndrome (MKS), Bardet–Biedl syndrome (BBS), and nephronophthisis (NPHP), have been categorized as rare diseases. However, recent findings have suggested that primary cilia play a significant role in the pathophysiology of various common diseases, such as cancers, diabetes, obesity, and atopic dermatitis (Liu et al., 2018; Hughes et al., 2020; Rizaldy et al., 2021; Xun et al., 2025). Consequently, ciliology has grown in significance as a field of basic and translational biomedical research.

**FIGURE 1:**
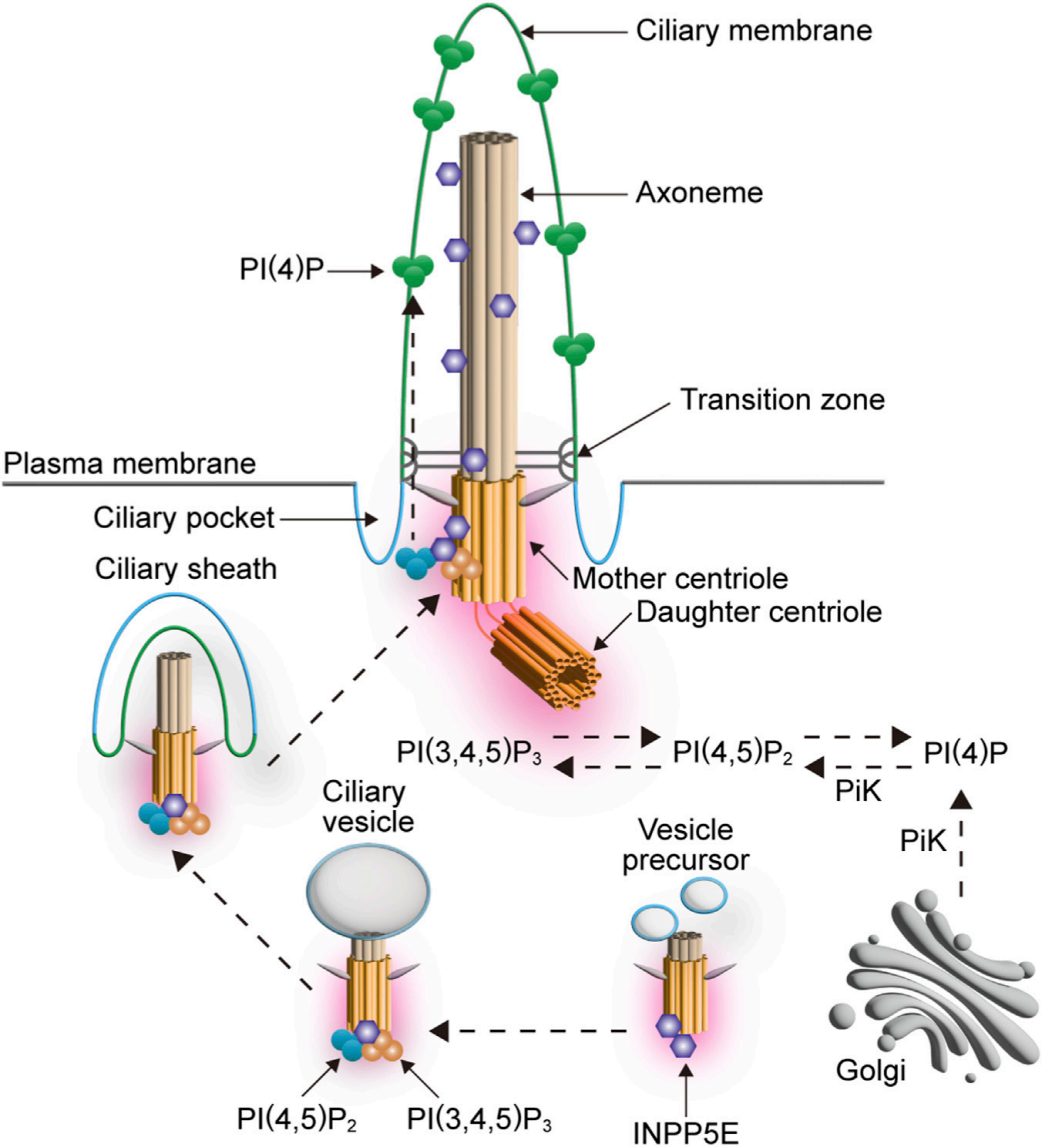
Regulation of phosphoinositide dynamics during ciliogenesis and ciliary maintenance. The schematic illustrates the molecular mechanisms governing phosphoinositide compartmentalization and cholesterol trafficking to the primary cilium. Ciliogenesis is driven by vesicle docking at the mother centriole, establishing a lipid gradient with PI(4)P concentrated in the ciliary membrane and PI(4,5)P_2_/PI(3,4,5)P_3_ restricted to the ciliary base and pocket.

The fundamental etiological mechanism of ciliopathies can be understood as an inability of primary cilia to sense the extracellular environment, resulting in the disruption of downstream signaling pathways. The primary ciliary membrane is enriched in a number of receptors mediating various signaling pathways, such as the Shh, Wnt, and PDGF pathways, as well as ion channels, such as TRP ion channels (Anvarian et al., 2019; Mill et al., 2023; Hansen et al., 2025). Thus, the membrane functions as a hub for information exchange between the intracellular and extracellular environments, serving as the point of origin for both extracellular environment detection and intracellular signal transduction. Thus, the primary cilia membrane containing unique proteins and lipids, does not simply serve as a passive structural boundary, but rather functions as a highly compartmentalized hub for receiving extracellular signals. To enable the sensitive detection of extracellular signals, the primary ciliary membrane contains unique protein and lipid components, rather than merely serving as a passive structural boundary essential for the maintenance of signaling fidelity and compartmentalization (Nechipurenko, 2020; Moran et al., 2024). Most of the previous cilia-related studies have mainly focused on the protein-based architecture of cilia, such as basal body proteins, TZ proteins, intraflagellar transport (IFT), and signal-transducing receptors. However, recently it has been shown that the lipid composition of the ciliary membrane is also a fundamental determinant of ciliary structure and function (Hilgendorf et al., 2024; Itabashi et al., 2026). For example, the ciliary membrane displays a highly distinctive phosphoinositide landscape, characterized by elevated levels of phosphatidylinositol 4-phosphate (PI(4)P) and exclusion of phosphatidylinositol 4,5-bisphosphate (PI(4,5)P_2_) (Figure 1) (Nakatsu, 2015). The maintenance of the phosphoinositide code in primary cilia relies on inositol polyphosphate 5-phosphatase (INPP5E), which is abundant in the base of the cilium and ciliary axoneme (Chavez et al., 2015; Garcia-Gonzalo et al., 2015). *INPP5E* is one of the genes responsible for JS, the product INPP5E has emerged as a central regulator of ciliary phosphoinositide identity by converting PI(4,5)P_2_ to PI(4)P. Loss of INPP5E allows aberrant PI(4,5)P_2_ accumulation within the ciliary compartment and disturbs the PI(4)P-enriched, PI(4,5)P_2_ signaling (Chavez et al., 2015; Garcia-Gonzalo et al., 2015). Recently, it was revealed that, in addition to phosphoinositides, sphingolipids, cholesterol, and lysolipids are also ciliary membrane components and are essential for cilia-associated signal transduction (Kaiser et al., 2020; Hu et al., 2021; Kimura et al., 2023). Thus, while the earlier review summarized the general roles of lipids in ciliary signaling (Nechipurenko, 2020), the rapid progress in “lipid ciliology” over the past few years demands an updated synthesis focusing on disease-specific mechanisms. Crucially, significant breakthroughs since 2020 have uncovered how precise lipid species directly gate ciliopathy-associated protein complexes. In this review, we explicitly build upon previous general overviews focusing on the newly emerged cholesterol–peroxisome–polycystin disease axis. We highlight recent structural and functional advances including peroxisome-dependent ciliary cholesterol delivery, sterol regulation of polycystin-2, and oxysterol-dependent polycystin activity, and discuss how these insights open novel therapeutic avenues for intractable ciliopathies like autosomal dominant polycystic kidney disease (ADPKD).

## 2 Intracellular lipid trafficking determines the unique ciliary membrane composition

Many kinds of lipids are biosynthesized in the endoplasmic reticulum (ER) and subsequently reach their target organelles through vesicular transport or organelle contact sites (Ikonen, 2008; Holthuis and Menon, 2014; Stefan et al., 2017). Vesicular transport is the classical mode by which lipids are moved between membrane-bound compartments along the secretory and endocytic pathways (van Meer et al., 2008). For this, lipids are incorporated into budding vesicles at a donor membrane and delivered to an acceptor membrane by vesicle fusion. This route is responsible for the bulk flow of lipids synthesized in the ER, including glycerophospholipids and sphingolipid precursors, through the Golgi apparatus and ultimately to the plasma membrane (van Meer et al., 2008). Membrane contact sites (MCSs) are regions where two organelle membranes are held in close apposition, typically within 10–30 nm, without fusing (Helle et al., 2013; Scorrano et al., 2019). At these sites, specialized protein complexes tether the opposing membranes and establish a microenvironment that permits direct lipid transfer between the bilayers, bypassing the need for vesicle budding and fusion. MCSs have now been documented between virtually every pair of intracellular organelles, including ER–plasma membrane, ER–mitochondria, ER–endosomes/lysosomes, and ER–Golgi contacts (Liou et al., 2005; Kornmann et al., 2009; Rocha et al., 2009). In general, lipid transfer proteins belonging to the STARD, ORP, and OSBP families mediate non-vesicular lipid transport at MCSs (Wong et al., 2019).

**FIGURE 2:**
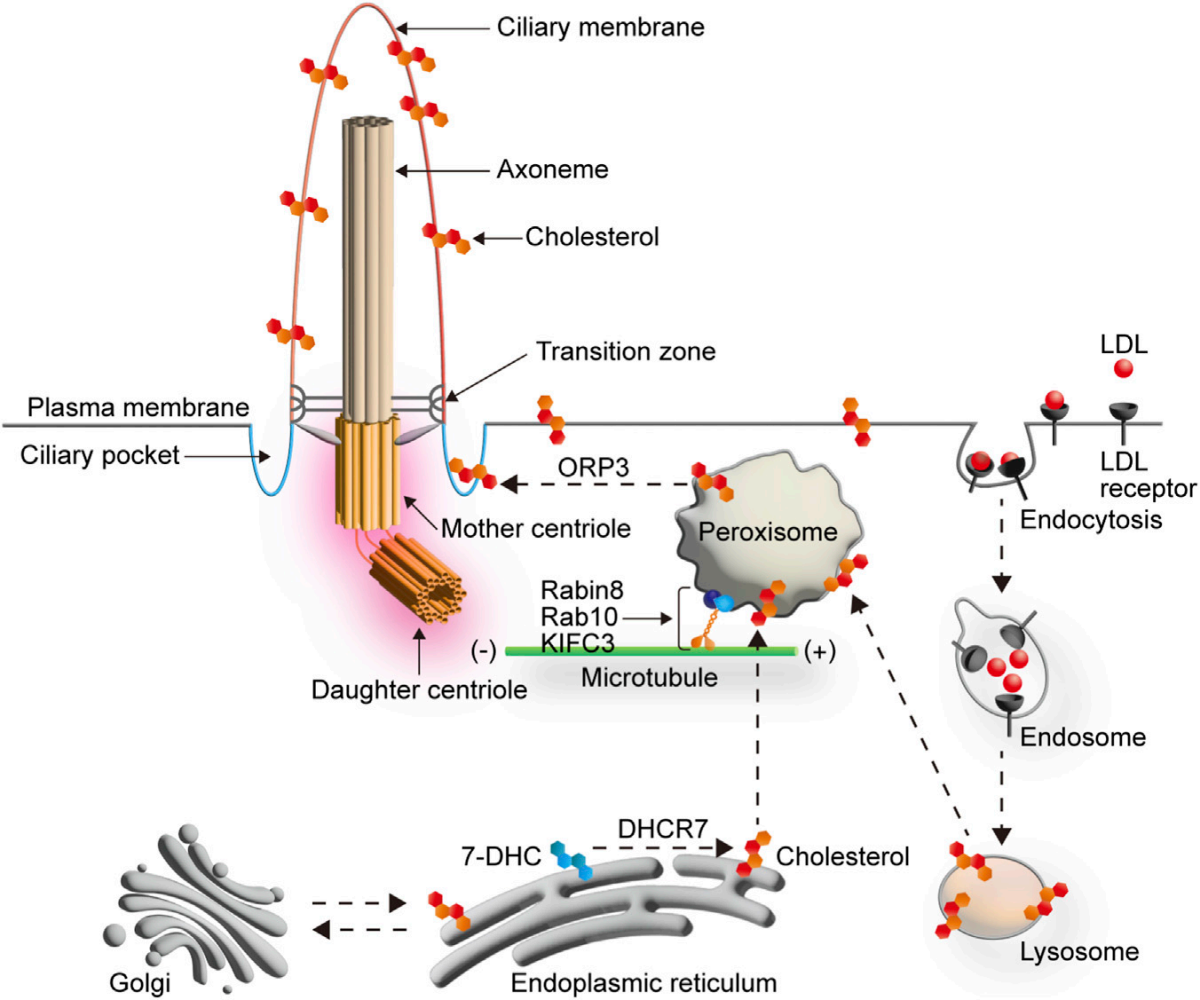
Intracellular cholesterol trafficking pathways to primary cilia. Ciliary cholesterol is supplied through coordinated inter-organelle pathways. *De novo* biosynthesis in the endoplasmic reticulum via DHCR7, LDL receptor-mediated endocytosis via lysosomes, and microtubule-dependent trafficking of peroxisomes regulated by the Rabin8–Rab10–KIFC3 complex and ORP3.

Primary cilia have been demonstrated to be rich in cholesterol (Kinnebrew et al., 2019; Miyamoto et al., 2020; Miyamoto et al., 2022). However, the mechanism underlying cholesterol transport has remained unclear for many years. We focused on the knowledge that peroxisomes are involved in intracellular cholesterol transport and that Zellweger syndrome, a peroxisomal dysfunction disorder, is associated with the development of polycystic kidney disease and retinitis pigmentosa, representing canonical manifestations within the ciliopathy spectrum (Chu et al., 2015; Braverman et al., 2016). Specifically, we hypothesized that peroxisomes serve as a source of cholesterol for the ciliary membrane and conducted analyses using cells from patients with Zellweger syndrome and cellular models. We found that peroxisomes move toward the centriole along microtubules via the Rabin8–Rab10–KIFC3 complex, forming MCSs between the peroxisomes and the ciliary pocket (Figure 2) (Miyamoto et al., 2020). We further found that cholesterol in the peroxisomal membrane is supplied to the ciliary membrane by ORP3, a cholesterol transporter present in the ciliary pocket (Miyamoto et al., 2020).

**FIGURE 3:**
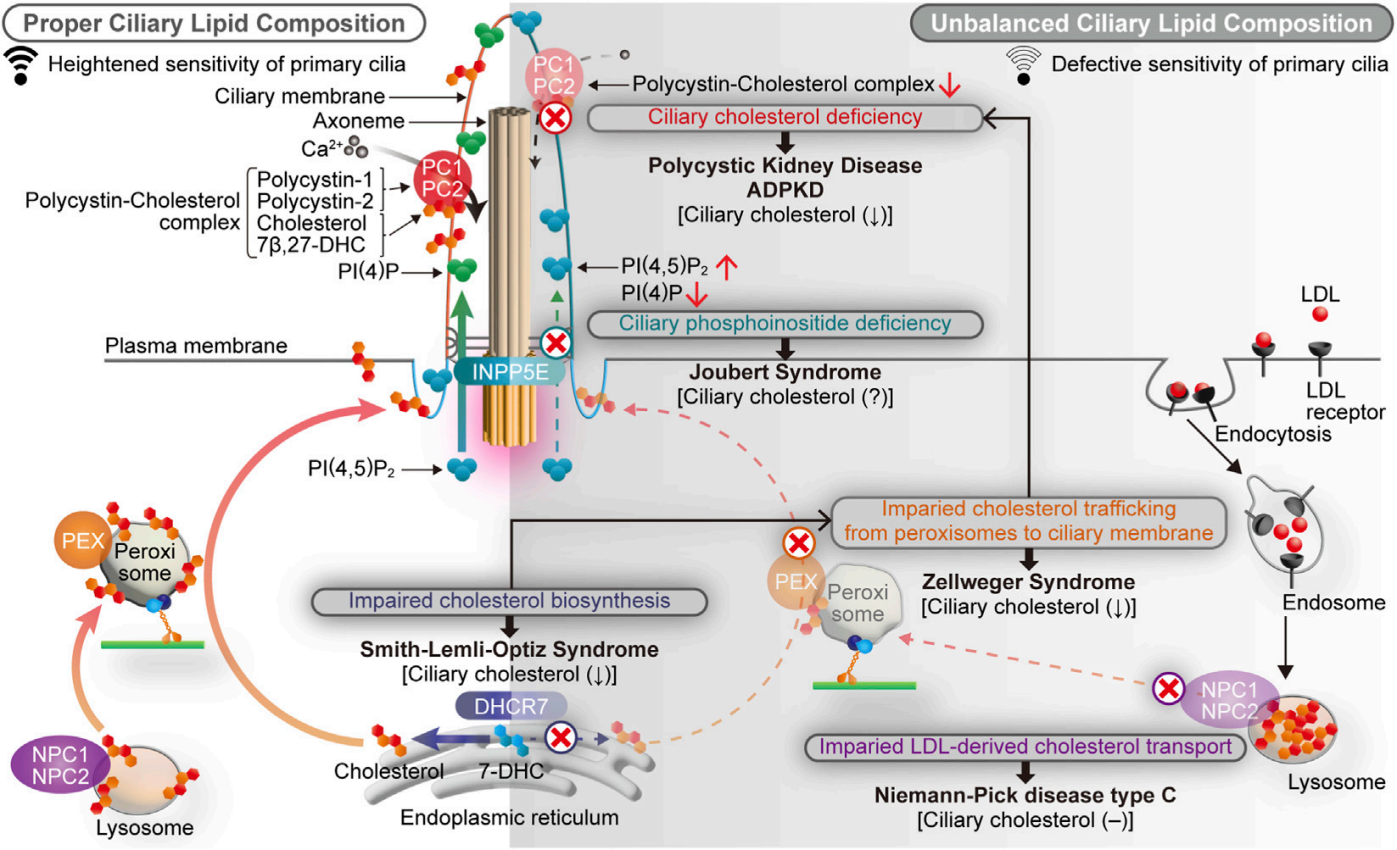
Impact of ciliary lipid imbalance on sensory function and associated ciliopathies. The schematic compares proper and unbalanced ciliary lipid compositions and their clinical consequences. In healthy cells, the primary cilium maintains elevated sensitivity through precise phosphoinositide compartmentalization (mediated by INPP5E) and coordinated cholesterol supply from the endoplasmic reticulum, lysosomes, and peroxisomes. This supports the functional assembly of the polycystin–cholesterol complex. Disruption of these lipid pathways (indicated by red crosses) leads to defective ciliary sensitivity and underlies various ciliopathies, including polycystic kidney disease, Joubert syndrome, Smith–Lemli–Opitz syndrome, Zellweger syndrome, and Niemann–Pick disease type C.

Cholesterol is known to be not only biosynthesized but also taken up from the extracellular space in the form of low-density lipoprotein (LDL) cholesterol (Figure 2) (Brown and Goldstein, 1986). LDL cholesterol is taken up into cells via endocytosis through LDL receptors (Maxfield and McGraw, 2004). Endosomes containing LDL cholesterol fuse with lysosomes and release esterified cholesterol. LDL receptors are then returned to the cell membrane via recycling endosomes, while cholesterol is transported from NPC2 in the lysosomes to NPC1 in the lysosomal membrane (Kwon et al., 2009; Li et al., 2016). Subsequently, cholesterol is transported via NPC1 to various organelles and the plasma membrane. A critical component in this distribution network is the dynamic formation of MCSs between lysosomes and peroxisomes, mediated by the interactions of lysosomal synaptotagmin VII with PI(4,5)P_2_ on the peroxisomal membrane (Chu et al., 2015). Through the created MCSs, LDL-derived cholesterol is transferred from the lysosomes to the peroxisomes. The functional importance of peroxisomes in this pathway is underscored by the observation that cells from patients with Zellweger syndrome, a peroxisome biogenesis disorder caused by *PEX* mutations, fail to deliver LDL-derived cholesterol to the ciliary membrane despite exhibiting normal lysosomal cholesterol release (Figure 3) (Miyamoto et al., 2020). These findings indicate that peroxisomes serve as an essential relay station for the ciliary delivery of both endogenously synthesized and exogenously acquired cholesterol. *NPC1* and *NPC2* are the causative genes for Niemann–Pick disease type C (NPC), an autosomal recessive disorder characterized by hepatosplenomegaly and neurodegeneration (Figure 3) (Carstea et al., 1997; Naureckiene et al., 2000; Vanier, 2010). In cells from NPC patients, free cholesterol accumulates in endosomes and lysosomes, leading to decreased cholesterol levels in the cell membrane (Chu et al., 2015; Miyamoto et al., 2020). Interestingly, however, the cholesterol levels in the ciliary membranes of NPC patient cells and *NPC1*-knockout cells are maintained at levels similar to those in normal cells, consistent with the clinical observation that most NPC patients do not develop typical ciliary disorders, such as polycystic kidney disease (Miyamoto et al., 2020). Moreover, the primary cilia in the hippocampus of *NPC1*-deficient mice are shorter and less responsive to Shh signaling (Canterini et al., 2017). However, the relationship between ciliary cholesterol levels and these *in vivo* alterations has not been investigated, and further research is needed. Based on the findings obtained to date, the peroxisome-mediated pathway responsible for supplying cholesterol to primary cilia is considered to be NPC1/NPC2-independent. Therefore, the altered ciliary features observed in NPC and Zellweger syndrome should be conceptualized as downstream, secondary consequences of systemic organelle dysfunction rather than primary defects of the ciliary apparatus itself. To provide conceptual clarity, it is valuable to adopt the classification proposed (Lovera and Luders, 2021). While “first-order” ciliopathies directly affect the structural or functional components of the cilium, metabolic syndromes like ZS (a peroxisome biogenesis disorder) and NPC (a lysosomal storage disease) are more accurately classified as “second-order” ciliopathies or “disorders with ciliary contribution.” In these conditions, the genetic defects primarily impair broad intracellular lipid trafficking and organelle crosstalk. This is supported by evidence showing that Zellweger syndrome patient cells display altered ciliary lipid distribution and compromised signaling pathways without showing gross morphological or numerical abnormalities in the cilia (Miyamoto et al., 2020), demonstrating that the ciliary phenotype arises secondarily to the disruption of the peroxisome-mediated lipid trafficking. Taken together, it has been revealed that endogenous and exogenous cholesterol in the ciliary membrane are supplied through a combined pathway involving transport by peroxisomes and MCSs between peroxisomes and cilia. Thus, intracellular lipid transport plays a crucial role in shaping the lipid landscape of the ciliary membrane, and disruptions in the associated pathways can lead to such second-order ciliary complications or metabolic syndromes with distinct ciliary contributions.

## 3 Ciliary and ciliary base localizations of lipid-related enzymes contribute to the unique ciliary membrane composition

The mechanisms underlying the unique lipid composition of the ciliary membrane include not only lipid transport but also regulation of the intracellular localizations of lipid synthesis and modifying enzymes to primary cilia and centrosomes (Nechipurenko, 2020). A well-characterized example of locally regulated lipid metabolism in primary cilia involves phosphoinositides (Chavez et al., 2015; Garcia-Gonzalo et al., 2015). The phosphoinositide 5-phosphatase INPP5E is specifically localized within the ciliary membrane, where it converts PI(4,5)P_2_ to PI(4)P, thereby establishing a phosphoinositide gradient that distinguishes the PI(4)P-enriched ciliary membrane from the flanking PI(4,5)P_2_-enriched plasma membrane (Figure 1). INPP5E carries a C-terminal farnesyl (prenyl) moiety i.e, recognized by the cytosolic chaperone PDE6D, which shields the hydrophobic group and ferries INPP5E across the TZ to the ciliary base (Humbert et al., 2012). Once inside the cilium, the activated form of the small GTPase ARL3, whose nucleotide exchange is promoted by the ciliary membrane protein ARL13B acting as a guanine nucleotide exchange factor, dissociates the PDE6D–INPP5E complex, releasing INPP5E into the ciliary membrane (Humbert et al., 2012). Notably, mutations in *INPP5E*, *ARL13B*, and *PDE6D* are individually causes of JS, underscoring the pathological importance of this enzyme-targeting machinery (Humbert et al., 2012).

A second layer of regulation involves cholesterol and oxysterol biosynthetic enzymes near the ciliary base. In general, cholesterol biosynthesis occurs in the ER, and 7-dehydrocholesterol reductase (DHCR7), which plays a role in the final stage of this process, is also located in the ER (Figure 3) (Luu et al., 2015; Koczok et al., 2019). Germline mutations in *DHCR7* cause Smith–Lemli–Opitz syndrome (SLOS), characterized by gonadal dysgenesis and mental retardation (Fitzky et al., 1998). It has been suggested that the clinical severity of SLOS is correlated with the degree of cholesterolemia (Jira et al., 2003). In severe SLOS cases, patients develop symptoms typical of ciliopathies, such as polycystic kidneys, polydactyly, and other skeletal abnormalities (Porter and Herman, 2011). Inhibition of cholesterol biosynthesis in zebrafish embryos by treatment with HMG-CoA reductase inhibitors (statins) causes visceral heterotaxy, a typical ciliopathy phenotype (Maerz et al., 2019). Clinically, statins show Shh signaling-related teratogenicity and are contraindicated in pregnant women (Incardona and Roelink, 2000; Edison and Muenke, 2004a; Edison and Muenke, 2004b). Although no structural abnormalities are detected in the primary cilia of skin fibroblasts derived from SLOS patients or statin-treated fibroblasts derived from healthy individuals, cilia-dependent Shh signaling is nonetheless impaired in these cells (Miyamoto et al., 2020). Based on these findings, SLOS is regarded as a ciliopathy caused by ciliary cholesterol deficiency, analogous to Zellweger syndrome (Miyamoto et al., 2020; Erickson and Fiorenza, 2026; Itabashi et al., 2026). Interestingly, DHCR7 is also localized in the vicinity of the ciliary base and has been shown to prime the ciliary membrane for Shh pathway activation (Findakly et al., 2021). In the Shh signaling pathway, Patched1, a receptor for the Shh ligand, becomes localized to primary cilia in the absence of the Shh ligand and regulates the amount of accessible cholesterol (free cholesterol not bound to sphingomyelin [SM]) in the outer leaflet of the ciliary membrane (Rohatgi et al., 2007; Kinnebrew et al., 2019; Kinnebrew et al., 2021). When the Shh ligand binds to Patched1, Patched1 dissociates from the primary cilium, and the seven-transmembrane protein Smoothened is recruited to the ciliary membrane. Activation of Smoothened at the ciliary membrane is thought to occur via the uptake of accessible cholesterol from the ciliary membrane. Specifically, the binding of cholesterol to the cysteine-rich domain located in the extracellular region of Smoothened induces its conformational change (Huang et al., 2016; Luchetti et al., 2016; Huang et al., 2018). Following the activation of Smoothened within the cilia, the Gli transcription factor is activated in the ciliary cytoplasm by processing. Subsequently, the Gli transcription factor translocates to the nucleus, where it triggers the expression of Shh signaling response genes (Haycraft et al., 2005; Humke et al., 2010). Upon Shh stimulation, DHCR7 is removed from the ciliary microenvironment, acting as a negative feedback mechanism that limits local cholesterol production once the pathway has been activated. Conversely, Shh signaling positively regulates the oxysterol synthase CYP7A1, which accumulates near the ciliary base and produces oxysterols that further amplify the pathway activation (Findakly et al., 2021). The enzyme HSD11β2, located in the ER, also contributes to oxysterol production (Raleigh et al., 2018). Endogenous oxysterols generated by HSD11β2, including 7β,27-dihydroxycholesterol (7β,27-DHC), are enriched within the ciliary membrane and directly activate Smoothened through its sterol-bound cysteine-rich domain (Raleigh et al., 2018). Thus, the subcellular localization of lipid-related enzymes to primary cilia is a key mechanism governing the composition of ciliary membrane lipids.

## 4 The transition zone maintains the unique lipid composition of the primary ciliary membrane

In the cell membrane, two distinct molecular domains need to be maintained in separation, a challenge that human cells have overcome through the evolution of specialized fence structures embedded within the lipid bilayer plane. Two well-characterized examples of such membrane fence structures are the epithelial tight junction and the neuronal axon initial segment (Tsukita et al., 2001; Rasband, 2010). The tight junction, positioned at the apical-most boundary of the lateral membrane, prevents epithelial plasma membrane proteins from diffusing laterally between the apical and basolateral membrane domains. Its fence function is mediated by the integral membrane proteins claudin and occludin, which form continuous strands that encircle the cell and physically restrict lateral diffusion within the lipid bilayer (Tsukita et al., 2001). The axon initial segment is a structurally distinct but functionally equivalent barrier that exists in neurons. It occupies the proximal portion of the axon, immediately distal to the cell body, and maintains a sharp boundary between the somatodendritic and axonal membrane domains. Its fence function depends on a dense submembranous scaffold built around ankyrin-G and βIV-spectrin, which anchors voltage-gated ion channels at the axon initial segment and simultaneously prevents somatodendritic transmembrane proteins from diffusing into the axon (Rasband, 2010).

The ciliary TZ represents a third example of this general principle. Located at the ciliary base between the basal body and the proximal axoneme, the TZ must maintain separation between the ciliary membrane, which is enriched in signaling receptors such as Smoothened, PDGFR-α, and GPCRs, and the surrounding plasma membrane of the cell body (Moran et al., 2024). Structural studies have shown that Y-shaped protein linkers connect the axonemal microtubules to the overlying ciliary membrane and rings of intramembranous particles, called the ciliary necklace, at the TZ, forming a physical scaffold at the membrane boundary (Gilula and Satir, 1972; Garcia-Gonzalo and Reiter, 2017). Associated protein complexes, including the MKS and NPHP modules encoded by ciliopathy genes, are concentrated in this zone and are thought to constitute the molecular machinery of the fence (Sang et al., 2011). Consistent with this, disruption of TZ components in model organisms leads to the mislocalization of ciliary membrane proteins, with receptors that normally reside exclusively within the ciliary membrane becoming redistributed to the plasma membrane at large (Williams et al., 2011; Jensen et al., 2015; Shi et al., 2017). Taken together, the TZ enables the ciliary membrane to maintain a unique biochemical environment in a domain i.e, distinct from other plasma membrane regions (Figures 1, 2).

## 5 The physiology and pathology of ciliary membrane lipids

This section explains the mechanism by which lipids accumulated in the ciliary membrane enable primary cilia function, as well as the diseases associated with primary cilia dysfunction and their pathophysiological mechanisms (Figure 3). To reconcile the diverse molecular mechanisms and clinical manifestations traversing lipid ciliology, the functional roles and disease associations of these distinct lipid classes, modifying enzymes, and intracellular transport systems are systematically cataloged in Table 1.

**TABLE 1:** Overview of lipid-regulating enzymes, transporters, and their ciliary roles in health and disease.

Gene name	Validated lipid-related ciliological functions	Hereditary diseases caused by germline mutation/OMIM_Phenotype MIM number	Typical clinical features			
			PKD	Retinopathy	Cerebellar atrophy	Situs inversus
*CYP7A1*	Production of oxysterol near the ciliary base to activate smoothened in the shh signaling pathway	N.D.	N.D.	N.D.	N.D.	N.D.
*DHCR7*	Cholesterol biosynthesis at the ER to maintain sufficient levels of cholesterol in the ciliary membrane	Smith-lemli-opitz syndrome/270,400	O	O	O	N.D.
*FFAR4*	Production of omega-3 fatty acids to elevate intraciliary cAMP levels at the primary cilia	Susceptibility to obesity/607,514	N.D.	N.D.	N.D.	N.D.
*HSD11β2*	Production of oxysterol (7β,27-DHC) at the ER to activate smoothened in the shh signaling pathway	Apparent mineralocorticoid excess/218,030	N.D.	N.D.	N.D.	N.D.
*INPP5E*	Conversion of PI(4,5)P_2_ to PI(4)P at the ciliary base to regulate the phosphoinositide composition of the ciliary membrane	Joubert syndrome type 1/213,300	O	O	O	N.D.
*KIFC3*	Cholesterol trafficking to the ciliary pocket via a complex with Rabin8 and Rab10 localized on the peroxisome	N.D.	N.D.	N.D.	N.D.	N.D.
*NPC1*	Intracellular cholesterol trafficking from lysosomes to various organelles and the plasma membrane to maintain ciliary length and shh signaling	Niemann pick type C1/257,220	N.D.	O	O	N.D.
*NPC2*	Intracellular cholesterol transport to NPC1 at the lysosome	Niemann pick type C2/607,625	N.D.	O	O	N.D.
*ORP3*	Cholesterol transport at the ciliary pocket from peroxisomes to the ciliary membrane	N.D.	N.D.	N.D.	N.D.	N.D.
*PEX1/PEX2/PEX3/PEX5/PEX6/PEX10/PEX11B/PEX12/PEX13/PEX14/PEX16/PEX19/PEX26*	Biogenesis of peroxisome which plays a role of supplying cholesterol into the ciliary membrane	Zellweger syndrome/214,100,614,866,614,882,202,370,614,862,614,870,614,920,614,859,614,883,614,887,614,876,614,886,614,872	O	O	O	N.D.
*PTCH1*	Regulation of cholesterol trafficking to control the amount of accessible cholesterol in the ciliary membrane	Basal cell nevus syndrome 1, holoprosencephaly 7, gorlin syndrome/109,400	N.D.	N.D.	N.D.	N.D.
*PKD1*	Mechanoreception at the ciliary membrane, detecting fluid flow and triggering extracellular Ca^2+^ influx via a complex with polycystin-2	Autosomal dominant polycystic kidney disease/173,900	O	N.D.	N.D.	N.D.
*PKD2*	Regulation of ciliary cholesterol level through cholesterol binding, and extracellular Ca^2+^ entry via a complex with polycystin-1 in the ciliary membrane	Autosomal dominant polycystic kidney disease/173,910	O	N.D.	N.D.	N.D.
*PI3KC2α*	Phosphoinositides metabolism at the ciliary base to regulate ciliary entry of polycysitn 2 b y activating Rab11–Rab8 GTPase cascade	Oculoskeletodental syndrome/618,440	N.D.	O	O	N.D.
*PIP5K*	Production of PI(4,5)P_2_ in the ciliary membrane to promote the ciliary localization of polycystin-2	N.D.	N.D.	N.D.	N.D.	N.D.
*PDE6D*	Transport of INPP5E from the cytoplasm to the primary cilia	Joubert syndrome type 22/615,665	O	O	O	N.D.
*Rabin8*	Cholesterol trafficking to the ciliary pocket via a complex with KIFC3 and Rab10 localized on the peroxisome	N.D.	N.D.	N.D.	N.D.	N.D.
*Rab10*	Cholesterol trafficking to the ciliary pocket via a complex with KIFC3 and Rabin8 localized on the peroxisome	N.D.	N.D.	N.D.	N.D.	N.D.
*SMO*	Cholesterol binding receptor in the ciliary membrane	Curry-jones syndrome, pallister-Hall-like syndrome/241,800	N.D.	N.D.	O	N.D.
*SPTLC1/SPTLC2*	*De novo* biosynthesis of ceramide to regulate ciliogenesis	Hereditary sensory and autonomic neuropathy type 1 (HSAN1)/162,400, 613,640	N.D.	N.D.	N.D.	N.D.

Abbreviations: N.D., not determined/not documented.

### 5.1 Phosphoinositides

Phosphoinositides are a family of low-abundance signaling lipids generated by reversible phosphorylation of the inositol headgroup of phosphatidylinositol at the 3-, 4-, and/or 5-hydroxyl positions, yielding seven biochemically distinct molecular species: three monophosphorylated isomers, PI(3)P, PI(4)P, and PI(5)P; three bisphosphorylated forms, PI(3,4)P_2_, PI(3,5)P_2_, and PI(4,5)P_2_; and one trisphosphorylated form, PI(3,4,5)P_3_. The interconversion of these species is dynamically regulated by phosphoinositide kinases and phosphatases in a spatially and temporally controlled manner, enabling phosphoinositides to function as compartment-specific molecular determinants that recruit effector proteins and define organelle identity (Balla, 2013). As described above, the ciliary membrane is defined by a PI(4)P-enriched, PI(4,5)P_2_-depleted environment i.e, actively maintained by INPP5E. The canonical ciliary phosphoinositide map, which governs specific kinase/phosphatase dynamics and underpins syndromic ciliopathies, has been comprehensively reviewed elsewhere (Conduit and Vanhaesebroeck, 2020). Although standard models focus heavily on this PI code regarding Hedgehog signaling and transition zone integrity, we offer a distinct, complementary perspective by cross-examining how this PI framework intersects with a cholesterol- and polycystin-centered disease axis. Recent structural and trafficking discoveries suggest that the ciliary dosage and gating of the polycystin complex are co-regulated by both sterols and phosphoinositides, thereby offering fresh mechanistic insights into cystogenesis beyond traditional PI-centric models. The establishment of a ciliary phosphoinositide identity begins before the cilium is structurally visible (Figure 1). Initiation of cilium assembly requires the recruitment and fusion of membrane-bound vesicles at the distal appendages of the mother centriole, a process dependent on the local generation of PI(3,4,5)P_3_ (Figure 1) (Franco et al., 2014). Subsequently, phosphoinositide 3-kinase C2α (PI3KC2α) orchestrates the ciliary entry of polycystin-2 by activating a Rab11–Rab8 GTPase cascade, which in turn suppresses mTOR and mitogen-activated protein kinase (MAPK) signaling to prevent aberrant cyst formation (Margaria et al., 2020). This PI3KC2α–Rab11–Rab8 axis thus links phosphoinositide metabolism at the nascent ciliary base to the suppression of polycystic kidney disease-associated pathways, providing a mechanistic bridge between phosphoinositide signaling during early ciliogenesis and the ciliopathy spectrum, including the retinopathy and cerebellar hypoplasia seen in JS patients. The integrity of the ciliary phosphoinositide landscape is also critically dependent on the structural and functional integrity of the TZ, and mutations in TZ-associated proteins constitute a major etiological basis for syndromic ciliopathies. MKS, JS, and NPHP arise from loss-of-function mutations in TZ scaffold components, including *TMEM67*, *TMEM218*, *TMEM231*, *CEP290*, *NPHP1*, and *NPHP4*, which are required to maintain the PI(4)P-enriched, PI(4,5)P_2_-depleted environment of the ciliary membrane (Garcia-Gonzalo et al., 2015; Van De Weghe et al., 2022). When the TZ integrity is compromised, PI(4,5)P_2_ invades the ciliary axoneme, TULP3 aberrantly traffics GPCRs into the cilium, and GPR161, a negative regulator of Hedgehog signaling, becomes persistently mislocalized to the ciliary membrane (Mukhopadhyay et al., 2013; Garcia-Gonzalo et al., 2015; Badgandi et al., 2017; Dyson et al., 2017). The downstream consequence is a collapse of Shh signaling pathway output, contributing to neurodevelopmental abnormalities, polydactyly, renal cysts, and other convergent ciliopathy phenotypes (Reiter and Leroux, 2017). Perturbation of this phosphoinositide balance, whether through *INPP5E* mutations, phosphoinositide kinase dysregulation, or TZ gate disruption, constitutes a shared mechanistic basis for the convergent defects in Shh-dependent patterning observed across the ciliopathy spectrum.

### 5.2 Cholesterol

**FIGURE 4:**
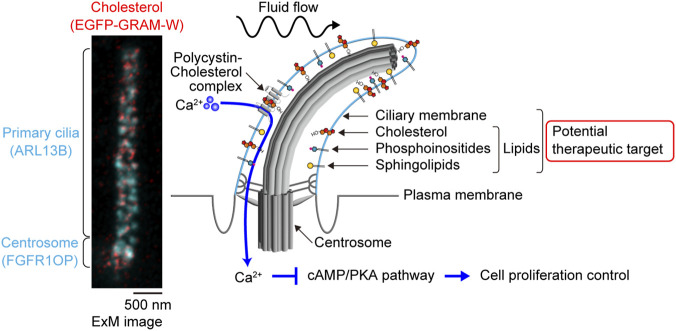
Ciliary membrane lipids as potential therapeutic targets (Left) Representative expansion microscopy (ExM) image of primary cilia. Quiescent G_0_-phase mouse collecting duct–derived (mIMCD3) cells transfected with EGFP-GRAM-W (red) as an accessible cholesterol probe (Koh et al., 2023) were immunostained with anti-ARL13B (cyan) and anti-FGFR1OP (cyan) antibodies. Scale bar, 500 nm. (Right) Schematic diagram of lipid composition and Ca^2+^ signaling in primary cilia. The ciliary membrane harbors distinct lipid domains enriched in cholesterol, phosphoinositides, and sphingolipids. In response to fluid flow, the polycystin-cholesterol complex activates Ca^2+^ influx, which in turn suppresses the cAMP/PKA pathway to control cell proliferation. Given this central role in ciliary signaling, ciliary membrane lipids represent a promising therapeutic target for cilia-related diseases.

Cholesterol has been established as a key ciliary membrane lipid that regulates Shh signaling. While disorders characterized by an insufficiency of ciliary cholesterol, such as SLOS and Zellweger syndrome, are associated with the development of polycystic kidney disease, cilia-related Shh signaling defects alone cannot fully explain the pathophysiological mechanisms underlying the polycystic kidneys (Ma et al., 2019). In recent years, a series of reports have described new mechanisms by which ciliary cholesterol prevents the development of polycystic kidney disease (Ha et al., 2024; Itabashi et al., 2025). Under physiological conditions, the primary cilia on renal epithelial cells detect urine flow to induce extracellular Ca^2+^ entry into the cytoplasm via a ciliary mechanosensitive ion channel complex, known as the polycystin complex, composed of the mechanosensor polycystin-1 and the TRP ion channel polycystin-2 (TRPP2) (Nauli et al., 2003; Bergmann et al., 2018). Ca^2+^ ions negatively regulate the cAMP/PKA pathway underlying cell proliferation and the luminal osmotic pressure (Figure 4), thereby maintaining the tubular lumen structure of the kidney (Bergmann et al., 2018). In polycystic kidney disease, reduced Ca^2+^ influx from primary cilia causes constitutive activation of the cAMP/PKA pathway, leading to expansion of the epithelial tubular lumen and the formation of cysts that become separated from the renal tubules (Bergmann et al., 2018). Germline mutations in *PKD1* and *PKD2* are responsible for autosomal dominant polycystic kidney disease (ADPKD) (Wilson, 2004). It has been proposed that the formation of multiple cysts in ADPKD patients follows a cellular recessive “two-hit” mechanism in which an inherited germline mutation in *PKD1* or *PKD2* is complemented by a secondary somatic mutation in the same gene within individual renal epithelial cells (Pei, 2001; Zhang et al., 2021). A combined analysis involving cryo-electron microscopy and molecular dynamics simulations revealed a cholesterol-binding site near the channel gate of the six-transmembrane protein polycystin-2 (Wang et al., 2020). The steroid nucleus of cholesterol is accommodated within a hydrophobic pocket formed by clusters of leucine (Leu517, Leu656), isoleucine (Ile561, Ile659), and valine (Val564, Val655) residues in polycystin-2. This pocket is located between the S3 and S4 helices of the voltage sensor-like domain and the S6 helix of the pore domain (Wang et al., 2020). Interestingly, the missense mutation L517R, which is part of the cholesterol-binding site detected in ADPKD patients, significantly reduced the binding of cholesterol to polycystin-2 (Wang et al., 2020; Itabashi et al., 2025). Although the *PKD2* L517R mutant retained its activity as an ion channel, its localization to primary cilia was significantly impaired (Itabashi et al., 2025). Furthermore, live-cell imaging of a polycystin-2–GFP fusion protein revealed that the removal of ciliary cholesterol by methyl-β-cyclodextrin treatment gradually reduced the ciliary signal of polycystin-2–GFP near the basal body, suggesting that polycystin-2 diffused laterally within the membrane (Itabashi et al., 2025). These observations suggest that ciliary cholesterol traps polycystin-2 within the primary cilium compartment, leading to the accumulation of the polycystin complex in the ciliary membrane. A comparison of the three-dimensional structures of wild-type polycystin-2 and the polycystin-2 L517R mutant protein is expected to elucidate the mechanism of cholesterol-mediated accumulation of the polycystin complex in cilia. It has been reported that polycystin-2 possesses distinct sterol-binding pockets formed by the pre-S1 helix and the linkers between S4 and S5, and that 7β,27-dehydrocholesterol (7β,27-DHC), an oxysterol, binds to this region and regulates ciliary ion channel activity (Ha et al., 2024). Moreover, the ciliary localization of polycystin-2 mutant proteins lacking 7β,27-DHC binding is not impaired (Ha et al., 2024). However, because the ciliary localization of the polycystin-2 mutants was analyzed in cells overexpressing polycystin-2, the findings may not accurately reflect the precise intracellular localization (Ha et al., 2024). Taken together, ciliary cholesterol functions as a factor that regulates the compartmentalization of the polycystin complex into the ciliary membrane and the activity of ion channels (Figure 3).

### 5.3 Sphingolipids

Sphingolipids are essential structural and signaling lipids that are abundant in the plasma membrane, particularly in lipid raft microdomains enriched with cholesterol and SM (Kaiser et al., 2020). Within the ciliary membrane, SM negatively controls the level of accessible cholesterol by sequestering it in SM–cholesterol complexes, thereby restraining Shh signaling under basal conditions. Depletion of SM increases accessible cholesterol in the ciliary membrane and potentiates Smoothened activation and Shh signal output (Kinnebrew et al., 2019). Ceramide plays a particularly important structural role in ciliogenesis through its participation in IFT-mediated axonemal organization. Global translatome profiling during cilia regeneration in *Chlamydomonas* identified serine palmitoyltransferase (SPT), the rate-limiting enzyme in *de novo* ceramide biosynthesis, as an essential regulator of ciliogenesis. Cryo-electron tomography revealed that loss of ceramide abnormally increased the distance between the ciliary membrane and the axoneme, generating bulging cilia, while ceramide–IFT particle–IFT motor–microtubule interactions physically connected the ciliary membrane with the axoneme to maintain the normal rod-shaped cilia morphology (Wu et al., 2022). Meanwhile, SPT-deficient human cells displayed defective ciliogenesis that could be restored by sphingolipid supplementation, further establishing a conserved and clinically relevant role for sphingolipids in cilia formation (Wu et al., 2022). The pathological relevance of SPT is highlighted by hereditary sensory and autonomic neuropathy type 1 (HSAN1), an autosomal dominant peripheral neuropathy caused by missense mutations in the *SPTLC1* or *SPTLC2* subunits of SPT (Rotthier et al., 2010). These mutations induce a shift in substrate specificity, favoring L-alanine over L-serine, leading to the accumulation of neurotoxic 1-deoxysphingolipids that cannot be converted to complex sphingolipids or degraded (Penno et al., 2010). HSAN1 patients exhibit progressive distal sensory loss, peripheral neurodegeneration, and, in some cases, progressive retinitis pigmentosa (Tuson et al., 2004).

Beyond phosphoinositides, cholesterol, and sphingolipids, a remarkably diverse array of other lipid classes also contribute to ciliary structure and signaling. The ciliary membrane harbors a unique lipidome i.e, compositionally distinct from the surrounding plasma membrane, encompassing phosphatidylethanolamine, ether-linked lipids, including plasmalogens, polyunsaturated fatty acids, and bioactive lipid mediators, such as prostaglandins (Nechipurenko, 2020). The transverse distribution of lipids across the two membrane leaflets is itself a critical structural parameter. Lipid flippases, specifically the P4-ATPase ALA2, are recruited from the plasma membrane to the nascent ciliary membrane during ciliogenesis in *Chlamydomonas*, where they actively translocate the conically shaped phosphatidylethanolamine from the outer leaflet to the inner leaflet, thereby generating the membrane curvature required for the cylindrical ciliary architecture. Loss of ALA2 results in misshapen ciliary membranes, delayed ciliogenesis, and shortened cilia; similarly, depletion of the corresponding flippase in mammalian cells reduces ciliation (Wang et al., 2025). Omega-3 polyunsaturated fatty acids add yet another dimension to this complexity. Docosahexaenoic acid (DHA), the most abundant polyunsaturated fatty acid in photoreceptor outer segment membranes, is essential for maintaining the membrane fluidity and biophysical properties that support rhodopsin activation and phototransduction in the connecting cilium and outer segment discs (SanGiovanni and Chew, 2005; Swinkels and Baes, 2023). Beyond its structural role, DHA acts as a ligand for the free fatty acid receptor FFAR4/GPR120, which is specifically concentrated in the primary cilia of preadipocytes in a TULP3-dependent manner. Ciliary activation of FFAR4 by DHA rapidly elevates intraciliary cAMP levels, triggering EPAC-dependent adipogenesis, thereby establishing omega-3 fatty acids as systemic metabolic signals decoded at the ciliary membrane (Hilgendorf et al., 2019). Furthermore, lipid-mediated post-translational modifications of ciliary proteins, including palmitoylation, myristoylation, and prenylation, are critical for ciliary protein trafficking and membrane anchoring, and their disruption leads to ciliopathy phenotypes (Roy and Marin, 2019). Taken together, these findings underscore that the ciliary membrane is not a passive lipid bilayer but a precisely orchestrated lipid landscape spanning dozens of molecular species, each playing distinct structural or signaling roles. Despite significant recent progress, the mechanisms by which cilia actively establish and maintain their unique lipidome against the continuous plasma membrane, how individual lipid species are spatiotemporally organized across the ciliary subdomains, and how their collective dysregulation drives the full spectrum of ciliopathies remain wide-open frontiers for research in the emerging field of lipid ciliology.

## 6 Future perspectives

The identification of ciliary membrane lipids as key regulators of ciliopathy-related protein complexes opens a new conceptual avenue in drug discovery for intractable diseases, a field we have designated lipid ciliology. ADPKD provides the most compelling proof-of-concept. The only globally approved disease-modifying therapy, tolvaptan, is a selective vasopressin V2 receptor antagonist whose efficacy in slowing cyst growth and preserving renal function was established by the landmark TEMPO 3:4 and REPRISE trials (Torres et al., 2012; Torres et al., 2017). Nevertheless, tolvaptan has some inherent limitations: its potent aquaretic mechanism invariably produces polyuria, polydipsia, and clinically significant risks of dehydration and hypernatremia (Chebib et al., 2025). Moreover, its safety and efficacy in dialysis patients, pregnant women, and children remain to be established, leaving a broad swath of patients without a targeted therapeutic option. These unmet needs have spurred several complementary pipelines. Tamibarotene (RN-014), a retinoic acid receptor agonist identified through induced pluripotent stem cell-based drug discovery, has demonstrated potent cyst-suppressing activity in murine ADPKD models and human kidney organoids and is currently in phase II clinical trials (Mae et al., 2023). However, the well-characterized teratogenicity of retinoid agonists will constrain its use in women of childbearing potential (Williams et al., 2020). A second emerging strategy targets miR-17, a microRNA that post-transcriptionally suppresses *PKD1* and *PKD2* in ADPKD (Lee et al., 2019). RGLS4326 and its next-generation successor RGLS8429 are oligonucleotide anti-miR-17 compounds that become preferentially distributed to the kidney and cyst epithelium, restoring polycystin expression and attenuating cyst growth in preclinical models (Lee et al., 2019). RGLS8429 is currently being evaluated in a phase 1b multiple-ascending-dose trial. Nevertheless, miRNA therapeutics face systemic challenges, including hepatotoxicity, the requirement for optimized kidney-selective delivery systems, and the burden of repeated parenteral dosing.

Against this backdrop, lipid ciliology offers a mechanistically distinct and potentially safer therapeutic framework (Figure 4). As demonstrated by recent studies, ciliary membrane cholesterol, supplied to the ciliary pocket by peroxisomes via ORP3-mediated lipid transfer, is required to retain the polycystin complex within the primary cilium (Miyamoto et al., 2020; Itabashi et al., 2025). These findings recast the historical empirical use of statins for ADPKD in a new and cautionary light (Torres, 2026). Although statins have long been prescribed for ADPKD with the rationale of improving renal hemodynamics through endothelial protection, data from randomized clinical trials have not consistently demonstrated benefits in slowing cyst growth or preserving renal function. From the perspective of lipid ciliology, this failure may be mechanistically intelligible. Statins reduce ciliary cholesterol in renal epithelial cells, which can disrupt retention of the polycystin complex in cilia and potentially exacerbate the ciliary dysfunction that underlies ADPKD pathogenesis. This paradox highlights a broader principle that drugs modulating bulk cellular lipid metabolism can exert unintended consequences on the ciliary lipidome, and that these consequences must be explicitly evaluated within the emerging framework of lipid ciliology. Interestingly, INPP5E deficiency or activation of type Iγ phosphatidylinositol-4-phosphate 5-kinase (PIPKIγ) decreases PI(4)P in the ciliary membrane and increases PI(4,5)P_2_. These changes promote the localization of polycystin-2 to cilia and lead to cyst suppression, suggesting that phosphoinositides contribute to the regulation of polycystin complex levels in the ciliary membrane (Chen et al., 2026). At first glance, this finding presents an intriguing tension with the canonical ciliary phosphoinositide code, which dictates that PI(4,5)P_2_ exclusion and PI(4)P enrichment are absolute prerequisites for maintaining ciliary identity and preventing signaling defects like those seen in Joubert syndrome (Conduit and Vanhaesebroeck, 2020). This apparent contradiction can be reconciled by distinguishing the lipid requirements for structural ciliary identity may fundamentally differ from the localized biochemical requirements for polycystin-2 sorting and channel gating. While systemic and persistent ciliary PI(4,5)P_2_ accumulation is undeniably detrimental to overall ciliary trafficking, a localized increase in PI(4,5)P_2_ may act as a potent polycystin-2-recruiting signal, potentially through direct charge-based interactions with the channel’s polybasic domains. Therefore, the therapeutic suppression of kidney cysts via PI remodeling does not simply override the canonical lipid code. Rather, it represents a context-specific compensatory mechanism whereby altering the lipid balance preferentially rescues polycystin complex dosage in the ciliary membrane, even at the temporary expense of the classic PI(4)P/PI(4,5)P_2_ ratio. In addition to ciliary cholesterol, phosphoinositides are expected to be the subject of future research and development as new therapeutic targets for ADPKD.

The therapeutic implications of lipid ciliology extend well beyond rare ciliopathies, such as ADPKD, to common diseases in modern medicine. Primary cilia on hypothalamic neurons and pancreatic β-cells function as metabolic sentinels. Ciliary dysfunction impairs leptin and insulin receptor signaling, and ciliopathy mouse models reliably develop obesity and type 2 diabetes before any peripheral metabolic derangement is evident, suggesting that subthreshold ciliary dysfunction may contribute to common metabolic diseases (Gerdes et al., 2014; Volta and Gerdes, 2017). The lipid raft dynamics at the ciliary base control insulin receptor recruitment to the cilium, and perturbation of these dynamics impairs adipogenic differentiation in mesenchymal progenitors (Nishimura et al., 2021; Yamakawa et al., 2021). In oncology, lipid ciliology offers an equally compelling but underexplored set of therapeutic opportunities. Although primary cilia are frequently lost in highly proliferative tumors, a clinically important subset of malignancies, including basal cell carcinoma, medulloblastoma, and gastrointestinal stromal tumors, retain functional primary cilia, which serve as platforms for oncogenic signal transduction (Hassounah et al., 2012; Kimura et al., 2023). Beyond the well-established role of ciliary cholesterol in Shh signal amplification, emerging evidence points to the Wnt/β-catenin pathway as an equally lipid-sensitive ciliary signaling axis. The Wnt co-receptor LRP6 has recently been shown to become localized to the ciliary membrane, and the subsequent β-catenin signalosome activation is critically dependent on its recruitment to cholesterol- and sphingolipid-enriched lipid raft microdomains (Bilic et al., 2007; Ozhan et al., 2013; Zhang et al., 2023). For example, targeting the ciliary lipidome by selectively depleting cholesterol from the ciliary membrane or disrupting lipid raft-dependent LRP6 signalosome assembly could represent mechanistically precise and spatially restricted strategies to suppress oncogenic Wnt signaling, with potentially fewer off-target effects than systemic pathway inhibitors. Taken together, these converging lines of evidence establish lipid ciliology not as a niche subdivision in cilia research but as a unifying framework for understanding how a precisely orchestrated lipid environment within an evolutionarily ancient organelle governs signaling fidelity across diverse cell types and disease contexts. As cilium-specific lipidomic platforms, spatially resolved cryo-electron tomography, and disease-relevant organoid systems continue to progress, the systematic mapping of disease-specific ciliary lipid signatures promises to yield a new generation of biomarkers and pharmacological targets for conditions that have resisted conventional therapeutic approaches against rare and common cilia-related diseases (Figure 4).

## 7 Conclusion

Lipid ciliology is not merely the study of lipids in cilia; it is the study of how life organizes chemical information at the nanoscale and the diseases that arise when this organization fails. In this review, we provide a systematic graphical framework (Figures 1–4) to conceptualize these dynamic processes. We visually charted the transition from physiological phosphoinositide codes (Figure 1) and multi-organelle cholesterol trafficking (Figure 2) to the pathological collapse of the ciliary lipid landscape (Figure 3). Furthermore, by coupling high-resolution expansion microscopy with signaling schematics (Figure 4), we underscored the basic biological and translational significance of targetable ciliary lipid domains.
